# Influence of Simulated Microgravity on Mammary Epithelial Cells Grown as 2D and 3D Cultures

**DOI:** 10.3390/ijms24087615

**Published:** 2023-04-20

**Authors:** Garrett Winkelmaier, Kosar Jabbari, Lung-Chang Chien, Peter Grabham, Bahram Parvin, Janice Pluth

**Affiliations:** 1 Electrical and Biomedical Engineering Department, University of Nevada, Reno, NV 89557, USA; 2 Epidemiology and Biostatistics Department, University of Nevada, Las Vegas, NV 89154, USA; 3 Center for Radiological Research, Columbia University, New York, NY 10032, USA; 4 Health Physics and Diagnostic Sciences Department, University of Nevada, Las Vegas, NV 89154, USA

**Keywords:** mammary, 3D organotypic culture, simulated microgravity, stem cell

## Abstract

During space travel, astronauts will experience a unique environment that includes continuous exposure to microgravity and stressful living conditions. Physiological adaptation to this is a challenge and the effect of microgravity on organ development, architecture, and function is not well understood. How microgravity may impact the growth and development of an organ is an important issue, especially as space flight becomes more commonplace. In this work, we sought to address fundamental questions regarding microgravity using mouse mammary epithelial cells in 2D and 3D tissue cultures exposed to simulated microgravity. Mouse mammary HC11 cells contain a higher proportion of stem cells and were also used to investigate how simulated microgravity may impact mammary stem cell populations. In these studies, we exposed mouse mammary epithelial cells to simulated microgravity in 2D and then assayed for changes in cellular characteristics and damage levels. The microgravity treated cells were also cultured in 3D to form acini structures to define if simulated microgravity affects the cells’ ability to organize correctly, a quality that is of key importance for mammary organ development. These studies identify changes occurring during exposure to microgravity that impact cellular characteristics such as cell size, cell cycle profiles, and levels of DNA damage. In addition, changes in the percentage of cells revealing various stem cell profiles were observed following simulated microgravity exposure. In summary, this work suggests microgravity may cause aberrant changes in mammary epithelial cells that lead to an increase in cancer risk.

## 1. Introduction

A major stressor in space for astronauts is exposure to microgravity. Previous studies reveal that the structure of cells is altered under microgravity, with cells exhibiting cytoskeleton reorganization [1,2], and changes in cell shape [3] and chromatin structure [4]. In addition, significant changes in protein expression related to cell adhesion and migration were noted in response to simulated microgravity (sim µg) exposure [5]. These morphological changes were also accompanied by changes in cell cycle profile, proliferation [6], apoptosis [7], and cellular signaling [8]. Owing to the unique function and nature of different cell types, it is likely that microgravity will impact the genetic, molecular, and biochemical mechanisms of different tissues and cell types uniquely. A majority of previously simulated microgravity cellular studies have focused on terminally differentiated cell lines or used cancer cell lines, which do not allow a complete understanding of the in vivo situation.

It is particularly important to understand how somatic stem cells, which have the ability to differentiate into a number of cellular lineages, will be affected by microgravity as changes in these cells could potentially cause more significant effects to organ function and development. Stem cells are of key importance for tissue regeneration following environmental exposures to such things as radiation, which can result in cell death. A small number of studies have focused on understanding the effects of microgravity on somatic stem cells; however, these studies have centered primarily on two types of stem cells, hematopoietic stem cells (HSCs) [9] and mesenchymal stem cells [10]. Thus, further studies are needed to better understand the unique response for other tissue types and their associated adult stem cell populations.

The mammary gland is particularly relevant given the increasing number of female astronauts, and thus it is important to understand how microgravity exposures in space may affect this tissue type. Previous studies with immortalized mammary epithelial cells, as well as other cell types, reveal that simulated microgravity exposure causes alterations in cell-to-cell interactions and can impact stem cell function [5]. Some work also suggests that microgravity exposure may cause changes that lead to increased cancer risk [11].

In the current study, we analyzed mouse mammary epithelial cells (HC11) following exposure to sim µg and observed changes in cell shape, size, and cell cycle profiles, with sim µg decreasing the size of cells and increasing the proportion of cells in the S phase. Apoptosis and levels of DNA double-strand breaks (DSBs) were increased in sim µg exposed cells as well. In addition, the ability of exposed cells to form proper 3D mammary acini structures was investigated. Cells exposed to sim µg, and sub-cultured to grow as 3D acini structures, revealed abnormal changes in normal acini characteristics. In total, the results predict that in vivo growth of mammary glands under microgravity may lead to aberrant growth and development and potentially increase cancer risk.

## 2. Results

### 2.1. Growth under Simulated Microgravity Causes a Reduction in Cell Diameter

**Figure 1:**
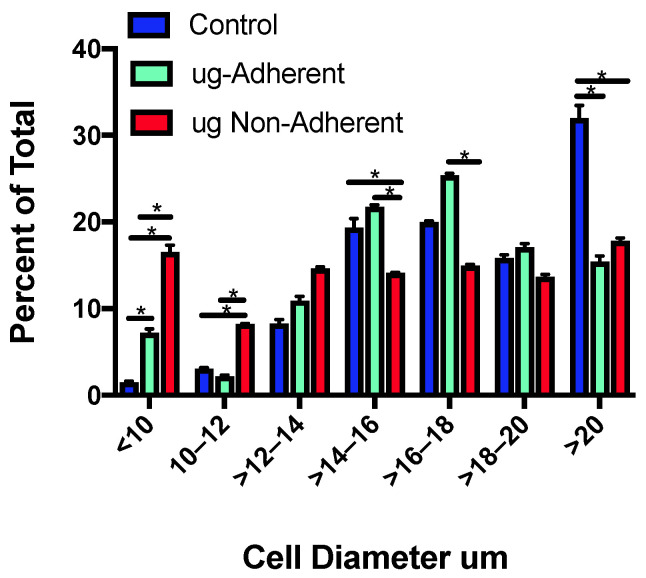
Binned cell diameters of control and simulated microgravity treated cells. The cell diameters of two populations of simulated microgravity cells (adherent and non-adherent) were defined, binned based on size and compared with controls (average % diameter +/− SE), * *p* < 0.004.

To define how sim µg exposure affected cell size, HC11 cells were seeded into T25 flasks and exposed to sim µg for 6 days. Roughly four times as many cells were seeded for sim µg flasks, as preliminary studies showed they tended to grow slightly slower as compared with controls. Six days after plating cells, control flasks experienced 8.8 population doublings, whereas the sim-µg-treated flasks only underwent 6.7 population doublings. During growth under sim ug, we also noted that a proportion of the cells remained adherent and a proportion detached and grew in solution. The population of cells that detached and grew in solution is herein referred to as the “non-adherent population”. Cell diameters were defined on a Cellometer (*n* = 513 for control, *n* = 362 for adherent, and *n* = 349 for non-adherent cells from two independent experiments), binned based on cell diameter (Figure 1) and compared for significant differences in binned sizes. Both the adherent and the non-adherent cells treated with sim µg showed a significantly greater number of small diameter cells as compared with control cells. In addition, a significantly smaller average diameter was observed in the sim-µg-treated non-adherent population as compared with the sim µg adherent population (average diameters: control = 17.4, adherent = 16.2, and non-adherent = 14.8). Control cells had only 4.5% of their population with a diameter under 12 µm, whereas the adherent population had 9% of their cells of this size and the non-adherent population had 25% of their cells with a diameter less than 12 µm. Control cells had significantly more larger cells, with ~twice the number of cells with a diameter over 20 µm as compared with the sim-µg-treated cells (32% as compared with 15% for the adherent and 17% for the non-adherent population).

### 2.2. Cell Cycle Profiles Are Altered after Simulated Microgravity Treatment

**Figure 2:**
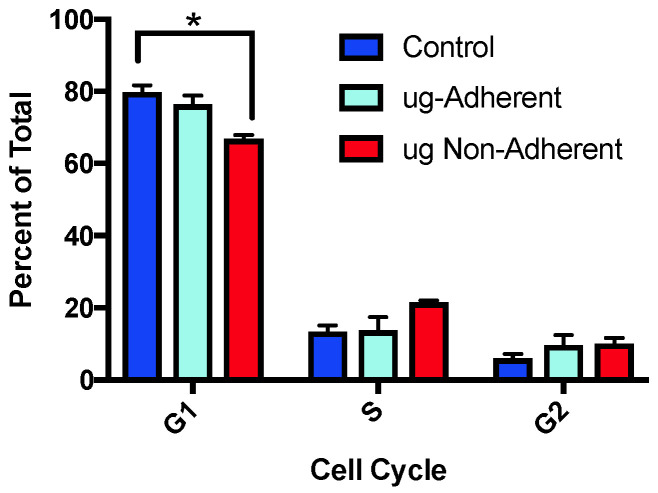
Differences in cell cycle kinetics when cells are grown under simulated microgravity. A significant difference in the percent of G1 cells was noted between control and sim-µg-treated adherent cells, * *p* = 0.02.

To define if sim µg exposure also altered cell cycle profiles, the percentage of cells in each cell cycle phase were defined. Cell cycle analysis on control and sim-µg-treated cells was performed using flow cytometry following propidium iodine staining. Two independent experiments were performed analyzing a total of 10,000 cells for each sample on a Guava 6HT-2L flow cytometer. The sim-µg-treated non-adherent cells had significantly less cells in G1 (Figure 2) as compared with the sim ug adherent population, with control cells showing similar G1 percentages as compared with the adherent cells. Correspondingly, the sim ug exposed non-adherent cells showed significantly higher levels of S phase cells as compared with controls, and G2 was slightly elevated, although not significantly in the sim-µg-treated cells as compared with controls (Figure 2).

### 2.3. Levels of γH2AX Differ with Sim µg Exposure

**Figure 3:**
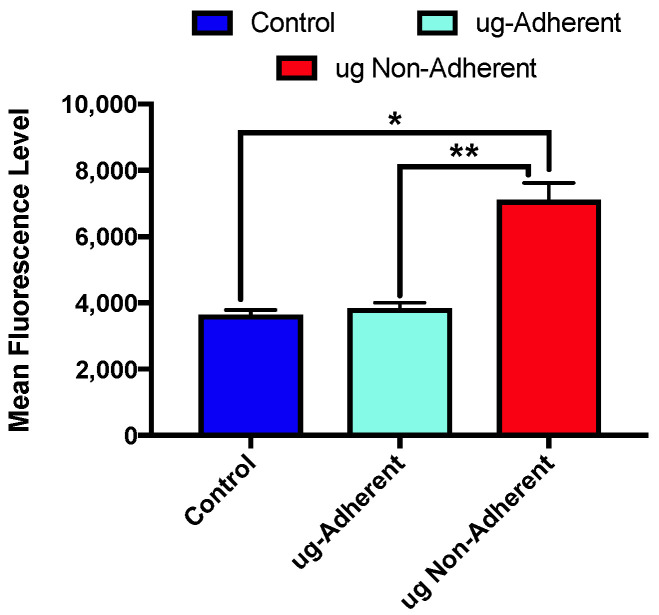
Levels of DNA double-strand breaks following sim µg exposure. HC11 cells were exposed to sim µg for 6 days and then assayed for levels of γH2AX using a flow-cytometry-based assay that defines average levels of fluorescence per cell; * *p* = 0.0002, ** *p* = 0.0009.

Given that previous work has shown that ROS is higher in microgravity treated cells and this may lead to DNA double-strand breaks (DSBs), we determined if DSBs were changed following the 6 days of sim µg exposure using a FACS-based γH2AX assay (Figure 3). Two independent sim µg exposures were performed and levels of γH2AX were quantified for the three populations of cells in replicate. Significantly higher levels of γH2AX were observed in the sim-µg-treated non-adherent cells as compared with both the control (*p* < 0.0006 control vs. non-adherent) and the adherent populations (*p* < 0.0009 adherent vs. non-adherent). The control and sim-µg-treated adherent cells showed fairly similar, lower levels of γH2AX.

### 2.4. Simulated Microgravity Causes an Increase in Apoptosis Specifically in Non-Adherent Cells

**Figure 4:**
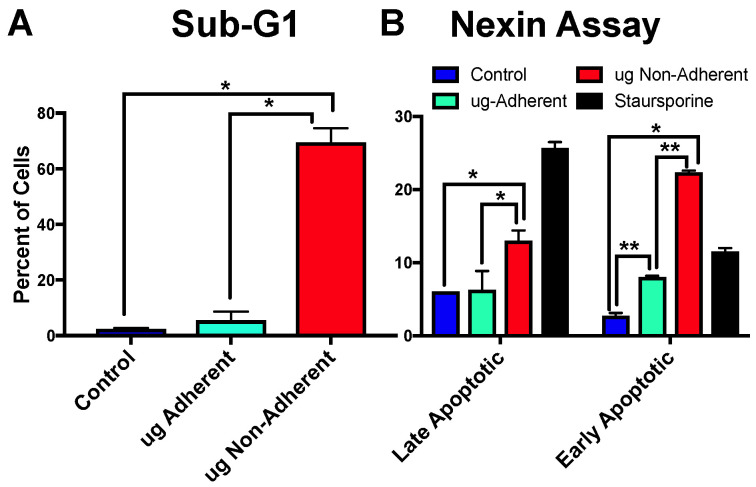
Levels of apoptosis as measured by sub-G1 apoptotic population of cells as defined by PI staining and flow cytometry (**A**), * *p* = 0.004. Early and late apoptosis as defined by the Nexin flow-based assay (**B**). Staursporine (black bar) was used as a positive control, * *p* ≤ 0.04, ** *p* = 0.0001.

Previous studies have also noted differences in apoptosis following sim µg exposure. Thus, we defined if the three populations (control, adherent, and non-adherent) of cells collected following 6 days of control or sim µg exposure had altered levels of apoptosis as measured by the sub-G1 peak of cells and the Nexin FACS-based assay. The percentages of sub-G1 cells were measured in two independent propidium iodide (PI)-stained experiments (Figure 4A) and significant differences were noted between control and non-adherent cells as well as between adherent and non-adherent cells. Early and late apoptosis was also measured in two additional independent experiments (Figure 4B) using the Nexin kit by Luminex (Austin, TX, USA). Staursporine was used as a positive control and, again, similar to the sub-G1 percentages, higher levels of both late apoptotic and early apoptotic cells were observed in the non-adherent sim-µg-treated cell populations. Thus, using both methods of analysis, higher levels of apoptosis were observed in the non-adherent cells exposed to sim µg as compared with control or sim-µg-treated adherent populations.

### 2.5. Growth under Simulated Microgravity Results in an Increase in a Small Sub-Population of Cells

**Figure 5:**
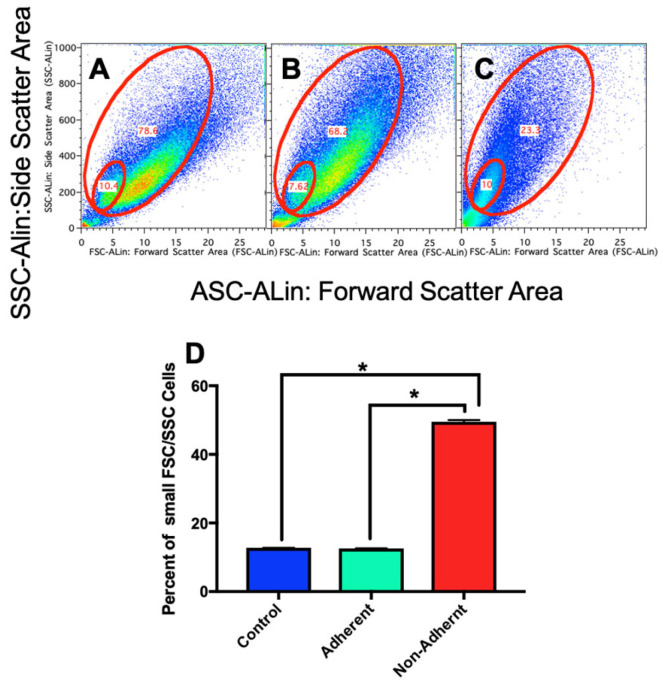
The percentage of small FSC vs. SSC population relative to the total in control (**A**) and sim-µg-treated adherent (**B**) and non-adherent (**C**) populations. The percentage of small cells based on FSC vs SSC in each of these groups is then graphed in (**D**) * *p* < 0.0002.

When performing FACS analysis, it was evident that a second population appeared to be increasing when gating on FSC (forward scatter) versus SSC (side scatter). Thus, we quantified changes in this sub-population of low FSC versus low SSC population following sim µg treatment. Cells were cultured under sim µg for 6 days and then assayed for changes in FSC versus SSC cell populations. The low FSC/low FSC population made up ~12% of the total population in control and adherent cells exposed to sim µg (Figure 5A,B). However, cells exposed to sim µg growing non-adherently appeared to lose much of the larger, high FSC/SSC population and have a greater percentage of the low FSC/low SSC population, representing ~50% of the total population of the cells (Figure 5C,D).

### 2.6. Stem Cell Populations Are Altered following Simulated Microgravity Exposure

**Figure 6:**
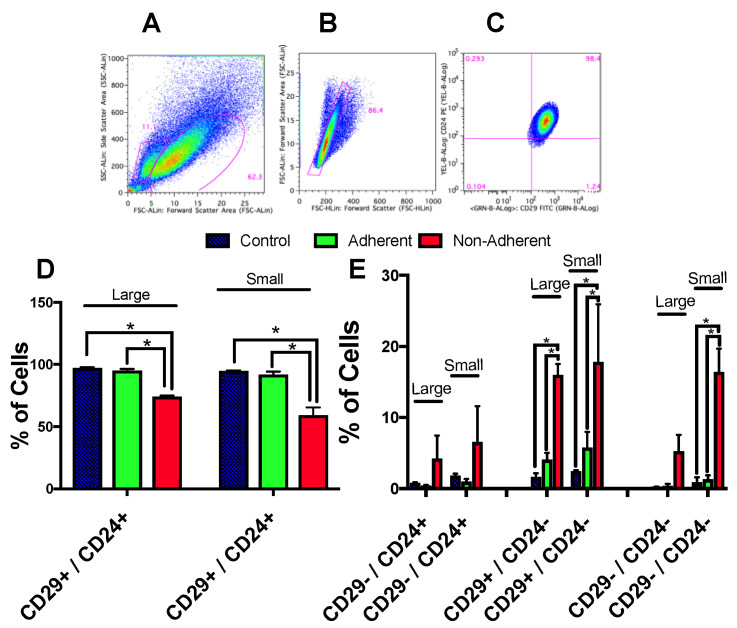
Stem cell markers altered following sim µg exposure. Based on FSC vs. SSC gating, two populations of cells were observed (**A**), with a smaller population falling lower on the FSC and SSC axis (small population). Doublet cells were eliminated (**B**) and the percentage of CD29 and CD24 positive or negative cells was analyzed (**C**). The majority of the population of treated and control cells (both large and small cells) were CD29+/CD24+; however, a decrease in this population was observed for sim-µg-treated non-adherent cells (**D**). In addition, higher numbers of CD29−/CD24+, CD29+/CD24−, and CD29−/CD24− cells were also noted in the non-adherent population, showing higher levels in the smaller population cells as compared with the larger population cells (**E**) * *p* < 0.05.

Given that this HC11 mammary cell line has been reported to have a higher percentage of stem cells, we wondered if these cells were changing under sim µg treatment. Prior to treatment, a majority of the control cells had CD24^High^/CD29^High^ markers, markers previous described by Shackleton et al., to identify mammary stem/stem cell progenitor populations [12]. Staining cells for these markers, we observed changes following sim µg exposure. Significant changes in the percentage of CD24−/CD29−, CD24− vs. CD29+, and CD24+/CD29+ cells were observed with sim µg treatment (Figure 6). When small and large populations based on FSC versus SSC were analyzed separately, a higher degree of change was noted in the small population of cells as compared with the large population of cells, with the non-adherent population showing the greatest changes in these markers as compared with the other two groups (control and sim-µg-treated adherent cells).

### 2.7. Three-Dimensional Acini Growth Is Altered with Simulated Microgravity Exposure

**Figure 7:**
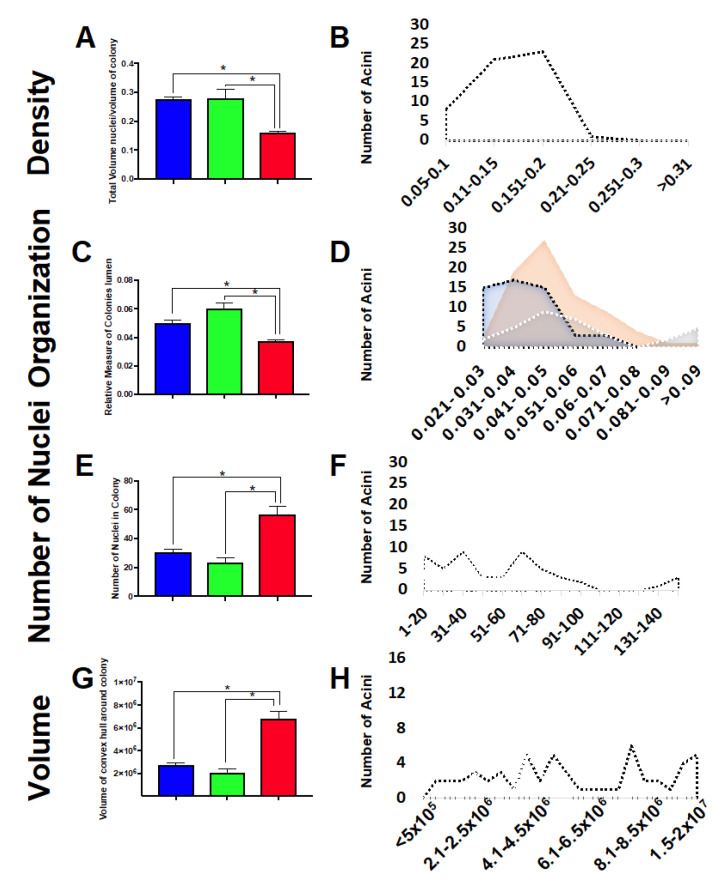
Three-dimensional acini growth changes quantified for control and simulated microgravity treated cell populations. Average values with standard error shown for each characteristic and group in (**A**,**C**,**E**,**G**). Values for each were binned to reveal in some cases sub-populations exhibiting varying degrees of each characteristic in (**B**,**D**,**F**,**H**), blue = control population, green = adherent population and red = non-adherent population of cells, (* *p* < 1 × 10^−5^).

To define if sim µg exposure affected the cell’s ability to properly form 3D structures, cells exposed to sim µg were set up in 3D cultures. Control as well as sim-µg-treated adherent and non-adherent cells were embedded into Matrigel independently and allowed to grow into 3D acini structures over a 6-day period. Then, 20× confocal images were taken of the stained acini, which were then analyzed using a computer software that non-subjectively defines differences in density, organization, proliferation, roundness, and volume (Figure 7). Significant differences were noted between non-adherent cells and both control and adherent cells in terms of density (Figure 7A,B), organization (Figure 7C,D), proliferation (Figure 7E,F), and volume (Figure 7G,H). Figure 7B,D,F,H show the binned cell populations, revealing that, in some cases, although the overall mean is similar for control and sim-ug-treated adherent cells, there are unique differences in sub-populations, with the sim-µg-treated population often showing a bimodal distribution, unlike the more bell-shaped population revealed in the control.

## 3. Discussion

Astronauts will be chronically exposed to microgravity over an extended period during a mission to Mars, or if man ever attempts to colonize other planets. Although human colonies in space seem a far-off possibility, understanding the effects microgravity may have on organ development is a critical step in understanding how potential future human colonies in space may be affected.

Regarding the effects of microgravity, many of the results of our study are consistent with the results from other researchers to date. Our studies have revealed that mouse mammary epithelial cells exposed to 6 days of sim µg develop changes in growth and cellular characteristics. In particular, cells that grow in solution while under sim µg exposure show the greatest degree of aberrant changes as compared with control cells. We observed an increase in smaller diameter cells and a decrease in G1 cells in the non-adherent sim-µg-treated cells. Research has shown that a number of signaling mechanisms, such as PI3K/Akt/mTOR, Myc, and hippo pathways, play a role in cell growth, cell size, cell division, and cell number as cells grow and divide [13]. During tumor development, these same pathways are often deregulated, causing changes in cell size and elevated proliferation. Stem cells have also been recognized to be of a smaller size as compared with differentiated cells in adult tissues, suggesting a relationship between small cell size and stemness [14]. In relation to our studies, the smaller size diameter cells within the non-adherent population could indicate a shift to more stem-like cells with sim µg exposure.

To define if the sim-µg-treated cells were also more negatively impacted in terms of DNA damage, we also measured DNA DSBs and apoptosis in the exposed cells as compared with controls. We observed an increase in apoptosis and DNA DSBs specifically in the non-adherent sim-µg-treated population of cells when compared with both control and sim-µg-exposed adherent cells. Work by Singh et al. [15] has suggested in human promyelocytic leukemia cells that ug induces DNA damage and mitochondria mediated apoptosis through the generation of ROS. Although we did not measure ROS levels in this work, others have published that microgravity increases the levels of ROS [16], perhaps indicating that sim µg exposure increased ROS, leading to increases in the DNA damage and apoptosis that we observed. However, it is surprising that only the sim-ug-treated non-adherent population revealed a significant increase in apoptosis and DNA DSBs.

Although the smaller diameter cells based on FSC/SSC levels that we observed with sim µg exposure may point to cells becoming more stem like, our experiments testing for changes in two mammary stem cell markers did not appear to indicate this. Instead, we observed a decrease in the numbers of cells with these particular stem cell markers in the non-adherent sim-µg-treated cells as compared with control and adherent sim-µg-exposed cells. Prior to sim µg exposure, control cells were primarily CD29+/CD24+, markers previously identified as markers of mammary stem/progenitor population. However, following exposure to sim µg, an increase in CD29−/CD24+, CD29+/CD24−, and double negative cells was observed. CD24+/CD29^lo^ positive cells have been shown to identify multipotent luminal progenitors and to give rise to mammary structures capable of differentiating, which cannot be serially transplanted [17]. Changes in these latter cell populations were the greatest in cells growing in solution under sim µg (non-adherent population). Thus, it is unclear what the changes in these cell markers indicate, there is a suggestion that they may lead to changes that alter morphology and proper stem cell balance.

Lastly, when sim-µg-treated cells are set up and cultured in 3D matrixes, cells that grew in suspension under sim µg were the most affected in terms of their ability to properly grow into 3D acini structures. Cells growing in suspension under sim µg and then placed into a 3D matrix to grow into acini show a decrease in density and organization, as well as an increase in the number of nuclei within the acini and an increased volume. These types of changes in cells growing non-adherently under sim µg show similarities to changes that arise in tumor cells cultured in 3D, which may indicate an increased risk for these cells to form tumors following sim µg exposure.

In summary, the findings of this work predict that organs in space may not properly form under microgravity and that their growth and development may be negatively impacted. In addition, the increase in proliferation and decreased organization suggests similarities to tumor phenotypes and may indicate that these sim-µg-treated cells may have a greater risk of promoting tumorigenesis. Obviously, these 3D acini are not complete organs, but rather mimic a sub-structure of the mammary gland that would form in vivo, thus further more in-depth microgravity studies using whole animals would be useful to verify these findings and additional studies using other organ types would be important to define the universality of this finding for other tissue types.

## 4. Materials and Methods

### 4.1. Cell Culture

HC11 mouse mammary epithelium cells were purchased from ATCC and used for these studies. HC11 cells are from a clone isolated from the parental cell line COMMA-1D. The Comma 1D parental cells were obtained from a pregnant mouse, are known to contain a high percentage of cells containing mammary stem or progenitor cell markers, and are able to self-renew and exhibit pluripotency [18]. They are capable of differentiation in culture without the addition of an extracellular matrix or co-culture with other cell types [18]. Cells were cultured in DMEM medium with 10% FBS, 1× antibiotic-antimycotic (Gibco, Billings, MT, USA), l-glutamine 4 mM), 5 µg/mL insulin, and 10 ng/mL EGF, under standard 5% CO_2_ and 37 °C. A total of 1000 or 4000 cells were plated per T25 flasks for control or simulated microgravity exposure, respectively. Cells were filled to capacity with medium and sealed with plugs such that no air bubbles were evident prior to placement on a 3D clinostat. The clinostat was run continuously for 3 days and then stopped for a medium change. Medium from simulated microgravity exposed flasks was spun down to collect any cells in suspension to return to flasks after feeding before exposing cells to another 3 days of simulated microgravity. Following a total of 6 days, cells were either analyzed directly (referred to as 2D experiments) or set up in Matrigel to allow for acini formation (referred to as 3D cultures), growing for an additional 6 days (see Section 4.8 for 3D acini growth and culture).

### 4.2. Three-Dimensional Clinostat (RPM)

**Figure 8:**
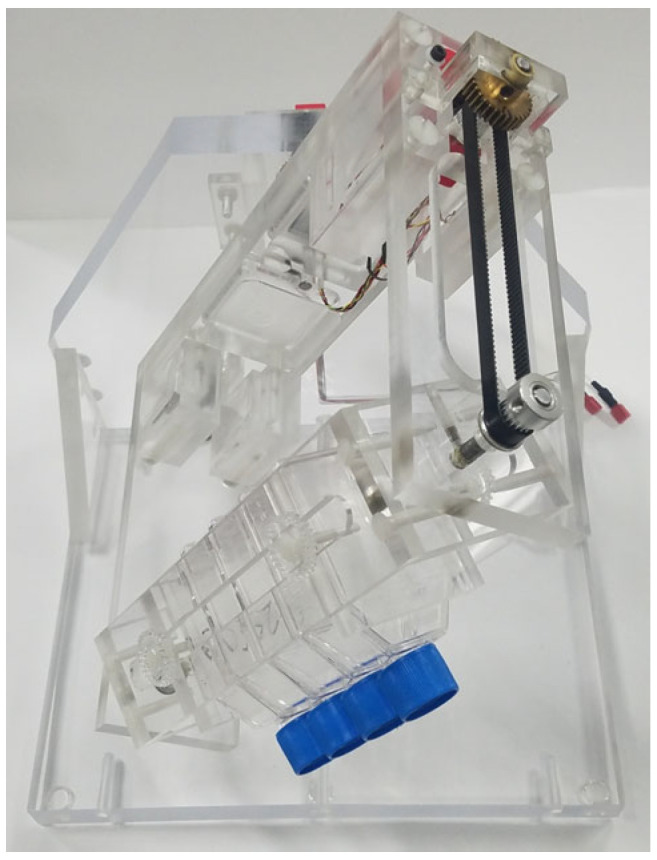
Three-dimensional clinostat used to simulate microgravity.

Microgravity conditions were simulated using a standard 3D clinostat, built in the UNLV engineering department based on plans received from Columbia University (Figure 8). The 3D clinostat rotates with a constant direction and speed under two rotation axes within each other and was operated in these studies at a speed of 1 RPM, providing a g = 10^−3^. During the 6-day exposure, flasks containing cells were placed within a standard incubator at 37 °C and the clinostat was connected to the controls through electrical cables running through the back of the incubator.

### 4.3. Cell Cycle and Sub-G1 Analysis 

A 6HT-2L Guava flow cytometer was used to define the percentage of cells in each cell cycle and the percentage of sub-G1 cells following propidium iodine staining. Flowjo software (Version 10.8.0 for Mac, Ashland, OR, USA) was used to define the percentage of sub-G1 cells and the percentage of cells in each cell cycle phase. A total of 20,000 cells were analyzed from each of two independent experiments. The control as well as adherent and non-adherent simulated microgravity cells were analyzed separately and significant differences between the defined groups noted.

### 4.4. Cell Diameter

Following control and six days of sim µg exposure, the cell diameter was analyzed using a Nexcelom Cellometer (Nexcelom Bioscience, Lawrence, MA, USA). Two independent experiments were performed and a total of 513 cells were analyzed for the control, as well as 362 for adherent cells and 349 for the non-adherent cells. Cells were binned based on size and significant changes with sim µg exposure defined.

### 4.5. γH2AX Levels

Following control and sim µg exposure, cells were washed in PBS, set up at 1 × 10^7^/mL, and fixed in 100% ice cold methanol. Then, 0.5 × 10^6^ cells were stained from control and sim-µg-exposed adherent and non-adherent cells. Cells were stained basically as described in Whalen et al. [19]. Briefly, cells were placed in a blocking solution containing 2% FBS/PBS and incubated for 1 h in primary antibody on ice. After incubation, cells were pelleted and washed in PBS and then incubated for 1 h on ice under foil with a secondary antibody diluted in blocking solution. Cells were then pelleted and washed for a final time in PBS. Cells were then resuspended in PBS containing PI and RNase or RNase alone to serve as a control for any spectral overlap of Molecular Probes 488 and PI. Two independent experiments were performed, collecting at least 10,000 cells per sample.

### 4.6. Apoptosis Assay

The Guava Nexin Reagent (Luminex, Austin, TX, USA) was used to define levels of apoptosis in control and sim-µg-exposed cells. The flow-based kit monitors the externalization of phosphatidylserine through the binding of Annexin V to the exposed phosphatidylserine. Normally, phosphatidylserine is localized to the inside of the cell membrane, but, during early apoptosis, it is translocated to the outer surface, where Annexin V can then bind to it. The Nexin reagent contains a PE-labeled Annexin to detect this early stage of apoptosis. 7-AAD is also contained in the kit, which will be excluded from healthy cells and only taken up by cells in late apoptosis. Thus, early and late apoptotic cells can be defined. Cells were processed and analyzed as per kit instructions.

### 4.7. CD24/CD29 Stem Cell Marker Profiling: Cell Labeling, Flow Cytometry, and Analysis

Cell labeling was carried out by staining with antibodies in buffer composed of PBS supplemented with 1% BSA. Cells were first suspended and placed in a blocking solution on ice at a concentration of 1 × 10^5^ cells in a total volume of 0.5 mL for 30 min and then stained with conjugated antibodies for 45 min on ice in the dark. After centrifugation at 250× *g* for 5 min at 4 °C, cells were washed with cold staining buffer before being subjected to flow cytometry. The antibodies used in this study were PE-conjugated CD 24 (BD Bioscience, Franklin Lakes, NJ, USA) and FITC-conjugated CD29 (Biolegend, San Diego, CA, USA). Proper isotype controls were used for each cell labeling experiment. The main cellular population was gated on FSC/SSC, then doublets were removed and the remaining cells were analyzed for percentages of cells showing CD29+/CD24+, CD29−/CD24+, CD29+/CD24−, or CD29−/CD24−. Flow cytometry was performed on a Guava 6HT-2L flow cytometer (Luminex, Austin, TX, USA) and data analyzed using FlowJo, LLC software (Mac Version 10.8.0 Ashland, OR, USA).

### 4.8. Three-Dimensional Acini Growth and Culture

**Figure 9:**
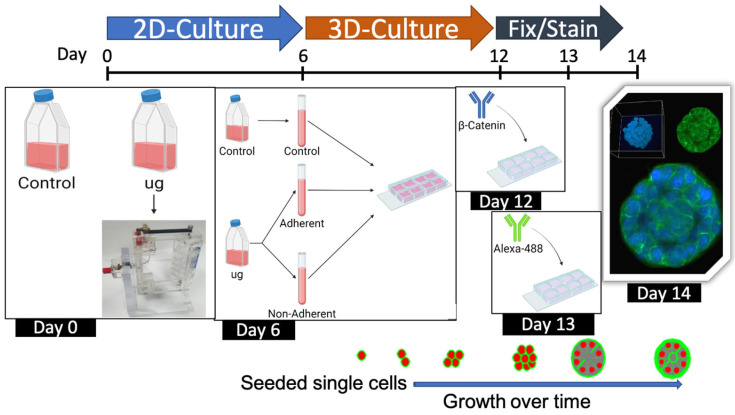
Overview of the culturing, treatment, and set up for both 2D and 3D cultures.

Following the exposure of cells to control or simulated microgravity, cells were counted and seeded into an eight-well chamber slide (Figure 9). Adherent and non-adherent simulated microgravity exposed cells were set up separately in individual wells for growth. The set up and culture for embedded acini growth was basically as described by Vidi et al. [20]. Briefly, Matrigel^TM^ (BD Biosciences, San Jose, CA, USA) was thawed overnight on ice at 4 °C on the day prior to set up. A small amount (7 µl) of chilled Matrigel was carefully distributed in an even layer into each well of a chamber slide sitting on a chilled block. Once evenly spread, it was placed into the incubator at 37 °C to solidify. Cells were trypsinized from the T25 flasks after growth under simulated microgravity exposure in 2D and then counted. Cells were diluted such that 175 cells were added to every 80 µL of Matrigel and this volume was layered on top of the previously plated layer of Matrigel. The slide was then placed in the incubator at 37 °C for 20 min and the chambers filled with medium and left to grow for a total of 6 days, changing the medium three days after set up.

### 4.9. Imaging and Analysis of 3D Acini Structure and Organization

Confocal images from each of the treatment groups were analyzed using a deep learning model optimized for characterizing 3D organoid models [21]. The number of images that could be captured were limited by the number of acini that formed following treatment for each group. From the control group, 53 images were analyzed; from the sim-µg-treated adherent group, 76 images were analyzed; and from non-adherent group, 37 images were analyzed. Briefly, to delineate each nucleus in a colony, we performed the method outlined in [21]. The number and position of nuclei, per colony, were used to compute indices in terms of the number of cells per colony and colony organization (e.g., elongation and roundness) per protocol outlined in [22]. A brief description of each colony structure follows. Volume was measured in voxels, and each image was converted to an isomorphic space where one voxel is equal to 1 cubic micron. Organization is a normalized value between 0 and 1, with 1 indicating that the colony is organized in a perfect sphere. Organization is unitless, as each nuclei’s distance to the colony center is divided by the maximum distance found. Density is the measure of all nuclear volumes found within a colony divided by the colony’s volume (i.e., convex hull around the colony), and this also is unitless. Number of nuclei is the number of nuclei in each acini colony, thus an absolute number.

### 4.10. Statistical Analysis

To define significant differences in each of the binned populations between the three groups (control, adherent, and non-adherent) in Figure 1, the Cochran–Mantel–Haenszel test was applied. For Figure 2, to compare if the percentages of cells in G1, S, or G2 were significantly different between the groups, an ANOVA was used (main effect: group; block effect: experiment). For Figure 3, to define if the mean γH2AX fluorescence levels were significantly different between groups, a repeated measure ANOVA with a randomized block design was used (fixed effect: group; block effect: experiment; repeated effect: replicate). For Figure 4A, to define if sub-G1 percentages were significantly different between the groups, an ANOVA was used (main effect: group; block effect: experiment). For Figure 4B, to define if significant differences were observed in necrotic, apoptotic, or viable cells, a repeated measure ANOVA with a randomized block design was used (fixed effect: group; random effect: experiment; repeated effect: replicate). For Figure 5, to define if the populations of any of the stem cell marker combinations (CD29−/CD24+, CD29+/CD24+, CD29+/CD24−, or CD29−/CD24−) were significantly different between the groups, for either the small or large cell size populations, a multivariate ANOVA (MANOVA) was used (main effect: group and size; interaction group × size) to account for the inter-correlations among the stem cell marker combinations. For Figure 6, to define if any of the characteristics of acini were significantly different (organization, convex hull-volume, density, number of nuclei, roundness) between the groups, the MANOVA approach was used (main effect: group) to account for the inter-correlations among the characteristics of acini. The rationale of utilizing MANOVA in Figure 5 and Figure 6 is that ANOVA assumes independence among the stem cell marker combinations or acini characteristics, which is not the case in this study. Lastly, to define if there was a significant difference in the percentage of large versus small cells (based on FSC vs. SSC), the ANOVA approach was used (main effect group and size; interaction group × size).

## Data Availability

Not applicable.
